# Amino Acid and Proliferation PET/CT for the Diagnosis of Multiple Myeloma

**DOI:** 10.3389/fnume.2021.796357

**Published:** 2022-01-06

**Authors:** Ryogo Minamimoto

**Affiliations:** Division of Nuclear Medicine, Department of Radiology, National Center for Global Health and Medicine, Tokyo, Japan

**Keywords:** multiple myeloma, ^11^C-4DST, ^18^F-FET, ^18^F-FLT, FDG, ^11^C-MET

## Abstract

Multiple myeloma (MM) is a hematologic malignancy characterized by infiltration of monoclonal plasma cells in the bone marrow (BM). The standard examination performed for the assessment of bone lesions has progressed from radiographic skeletal survey to the more advanced imaging modalities of computed tomography (CT), magnetic resonance imaging (MRI), and positron emission tomography/computed tomography (PET/CT). The Durie–Salmon PLUS staging system (upgraded from the Durie–Salmon staging system) applies 2-[^18^F]-fluoro-2-deoxy-glucose (^18^F-FDG) PET/CT, and MRI findings to the staging of MM, and ^18^F-FDG PET/CT has been incorporated into the International Myeloma Working Group (IMWG) guidelines for the diagnosis and staging of MM. However, ^18^F-FDG PET/CT has significant limitations in the assessment of diffuse BM infiltration and in the differentiation of MM lesions from inflammatory or infectious lesions. The potential of several new PET tracers that exploit the underlying disease mechanism of MM has been evaluated in terms of improving the diagnosis. L-type amino acid transporter 1 (LAT1), a membrane protein that transports neutral amino acids, is associated with cell proliferation and has strong ability to represent the status of MM. This review evaluates the potential of amino acid and proliferation PET tracers for diagnosis and compares the characteristics and accuracy of non-FDG tracers in the management of patients with MM.

## Introduction

Multiple myeloma (MM) is a clonal plasma cell proliferative disorder characterized by primary infiltration of bone marrow (BM), disruption of bone homeostasis, and excessive production of abnormal immunoglobulin that leads to bone destruction and marrow failure (1). Patients with suspected MM can be initially diagnosed with monoclonal gammopathy of undetermined significance (MGUS), solitary plasmacytoma, smoldering MM (SMM), or active (symptomatic) MM on the basis of clinical assessment. MGUS is the premalignant stage of MM, with an incidence of progression to MM of 0.5–1% per year. SMM is an intermediate clinical stage with a risk of progression to MM of 10% per year (2). In general, patients with SMM have almost no typical symptoms related to active MM, and no treatment is required. Confirmation of the MM status has implications in deciding the appropriate therapy.

2-[^18^F]-fluoro-2-deoxy-glucose (^18^F-FDG) positron emission tomography (PET)/computed tomography (CT) is now fully incorporated in the updated diagnostic and staging criteria for MM of the International Myeloma Working Group (IMWG) (3). According to the National Comprehensive Cancer Network (NCCN) guidelines, either whole-body low-dose CT or ^18^F-FDG PET/CT is recommended for the initial diagnostic workup for patients with suspected MM (4). There are several advantages of ^18^F-FDG PET/CT. It enables assessment of the tumor burden and disease activity of MM with high sensitivity and specificity (5). Patients with SMM have a high risk of progression to active MM if the lesions have positive ^18^F-FDG uptake (6, 7). ^18^F-FDG PET/CT can identify possible progression in patients with SMM and may have potential in decision-making for therapeutic interventions (7).

However, two significant limitations of ^18^F-FDG PET/CT are its poor sensitivity in patients with diffuse BM infiltration and its unreliable specificity due to its inability to discriminate between inflammatory/infectious lesions and MM lesions (8). Diffusion-weighted MRI identified viable disease in 11% of patients who were false-negative on ^18^F-FDG PET due to low hexokinase 2 (HK2) gene expression (9). Over a third of intramedullary myeloma lesions were undetected by ^18^F-FDG PET (10). For these reasons, alternative PET tracers that follow the molecular mechanism and disease condition of MM are under investigation. In this review, the potential of amino acid and proliferation PET/CT for the diagnosis of MM is discussed.

## Basic Mechanism of Amino Acid Transport in MM

The L-type amino acid transporter 1 (LAT1) is a membrane protein that transports neutral amino acids, functioning as a part of system L that provides nutrients for cellular intake (11, 12). LAT1 (4F2 light chain) is connected to the membrane-spanning 4F2 heavy chain (CD98) for its functional expression as part of a heterodimeric complex in the plasma membrane (12–14). Amino acid transporters (including LAT1) that intake essential amino acids as nutrients for cancer cells play a key role in cell growth and proliferation (11, 12, 15). The expression levels of LAT1 positively correlate with cell proliferation (Ki-67 labeling index), p53 expression, and vascular endothelial growth factor (VEGF) levels, as well as poor prognosis in various solid cancers (16, 17).

Isoda et al. (18) investigated the expression of LAT1 in patients with MM in an immunohistochemical staining study. They set three grades for the staining results (grade 0, no staining or <10% of tumor cells; grade 1+, ≥10% of tumor cells with weak staining intensity; grade 2+, ≥10% of tumor cells with moderate staining intensity; and grade 3+, ≥10% of tumor cells with strong staining intensity) and defined scores of 0 and 1+ as immunohistochemically low expression and scores of 2+ and 3+ as immunohistochemically high expression. High expressions of LAT1 and CD98 were detected in 56 and 45% of patients with MM, respectively. The expression score of LAT1 was positively correlated with the Ki-67 index (*r* = 0.631). Regarding Durie–Salmon stage (DSS), a statistically significant difference in the expressions of LAT1 and CD98 was confirmed between DSS I and II, and III. In contrast, age (≤65 or >65 years), sex, International Staging System (ISS), cytogenetic abnormality, extramedullary plasmacytoma, and BM plasma cells had no association with LAT1 or CD98.

The expressions of LAT1 and CD98 can predict 3-year progression-free survival (PFS) with hazard ratios higher than those of ISS, DSS, and cytogenetic abnormality. LAT1 is also a predictive marker of 3-year overall survival (OS) almost equivalent to ISS. The overall response rate in MM patients treated with melphalan and prednisolone was approximately 4 times higher in the high-LAT1-expression group than that in the low-LAT1-expression group. LAT1 was significantly associated with high proliferation and poor prognosis in newly diagnosed MM patients. These results indicate that LAT1 is a promising pathological marker for identifying high-risk MM (18).

## Basic Study of MET PET in MM

The major radiolabeled amino acid PET tracer is l-[*methyl*-^11^C]methionine (^11^C-MET), which can be rapidly synthesized in high radiochemical yield without the need for purification steps (19). The uptake of ^11^C-MET primarily indicates transmembrane transport majority by LAT1, which is influenced by the intracellular metabolism of the amino acid (20).

The levels of ^11^C-MET differ according to the myeloma cell line. ^11^C-MET uptake was significantly higher in cell lines characterized by higher levels of intracellular immunoglobulin light chains and higher CD138 and CXCR4 expressions on the cell surface, and the presence of cytogenetic aberrations was associated with worse prognosis (21–23). Based on the myeloma cell lines and patient-derived CD138^+^ plasma cells, retention was from 1.5- to 5.0-fold higher in ^11^C-MET than in ^18^F-FDG, even as early as 5 min post-tracer application (23).

As ^11^C-MET uptake can reflect myeloma tumor biology, it therefore has potential for the assessment of myeloma heterogeneity and the discrimination of tumor subtypes.

Treatment with proteasome inhibitors reduced the uptake of both ^11^C-MET and ^18^F-FDG in myeloma cell lines, and changes in tracer retention were associated with CD138 expression. In xenotransplant mice, ^11^C-MET uptake significantly decreased (by 30–79%) as early as 24 h after treatment; in contrast, ^18^F-FDG uptake showed no specific trend early after the treatment. This finding was confirmed in patient-derived MM cells. Reduction of ^11^C-MET uptake at 24 h after treatment initiation correlated with a reduction in tumor burden after completion of one cycle of chemotherapy and survival in mice. Thus, ^11^C-MET-PET has predictive potential regarding response and survival early after treatment initiation (24).

## Clinical Value of ^11^C-MET-PET in MM

**Figure 1:**
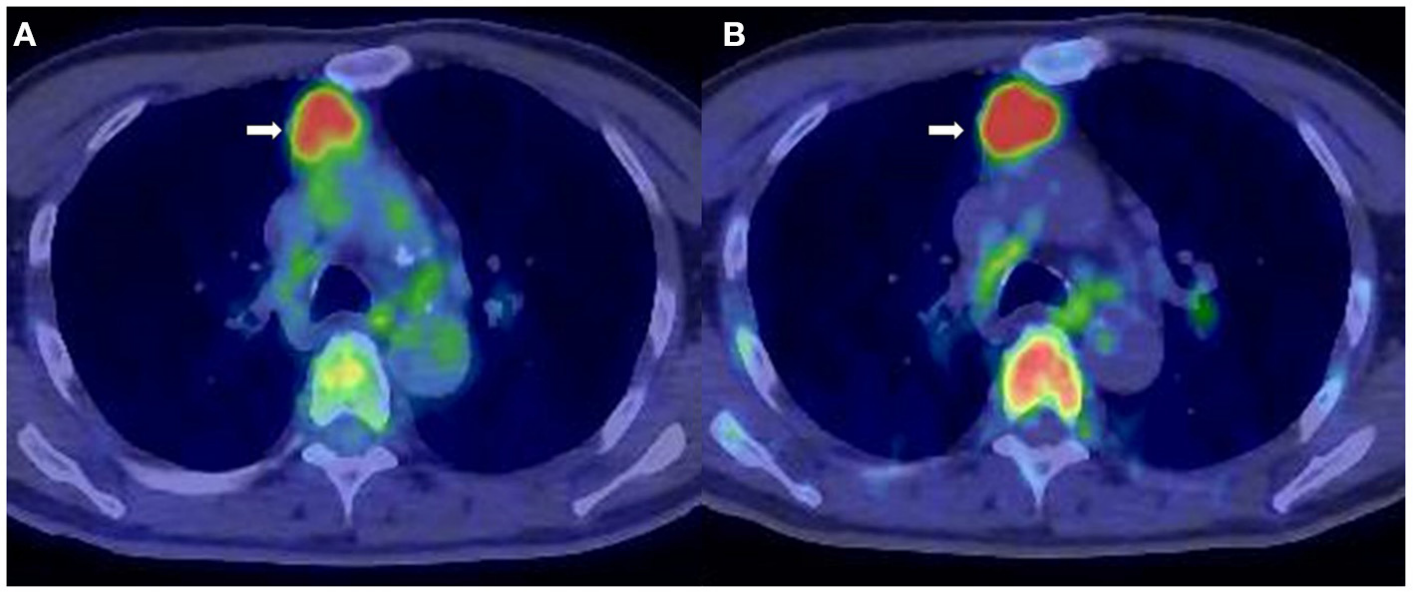
A 71-year-old man diagnosed with smoldering multiple myeloma (SMM). **(A)** 2-[^18^F]-fluoro-2-deoxy-glucose positron emission tomography/computed tomography (^18^F-FDG PET/CT). **(B)** l-[*Methyl*-^11^C]methionine (^11^C-MET) PET/CT. Extramedullary lesion in the mediastinum (*arrow*) was identified both by ^18^F-FDG PET/CT (SUV_max_ = 4.5) and ^11^C-MET PET/CT (SUV_max_ = 7.5).

**Figure 2:**
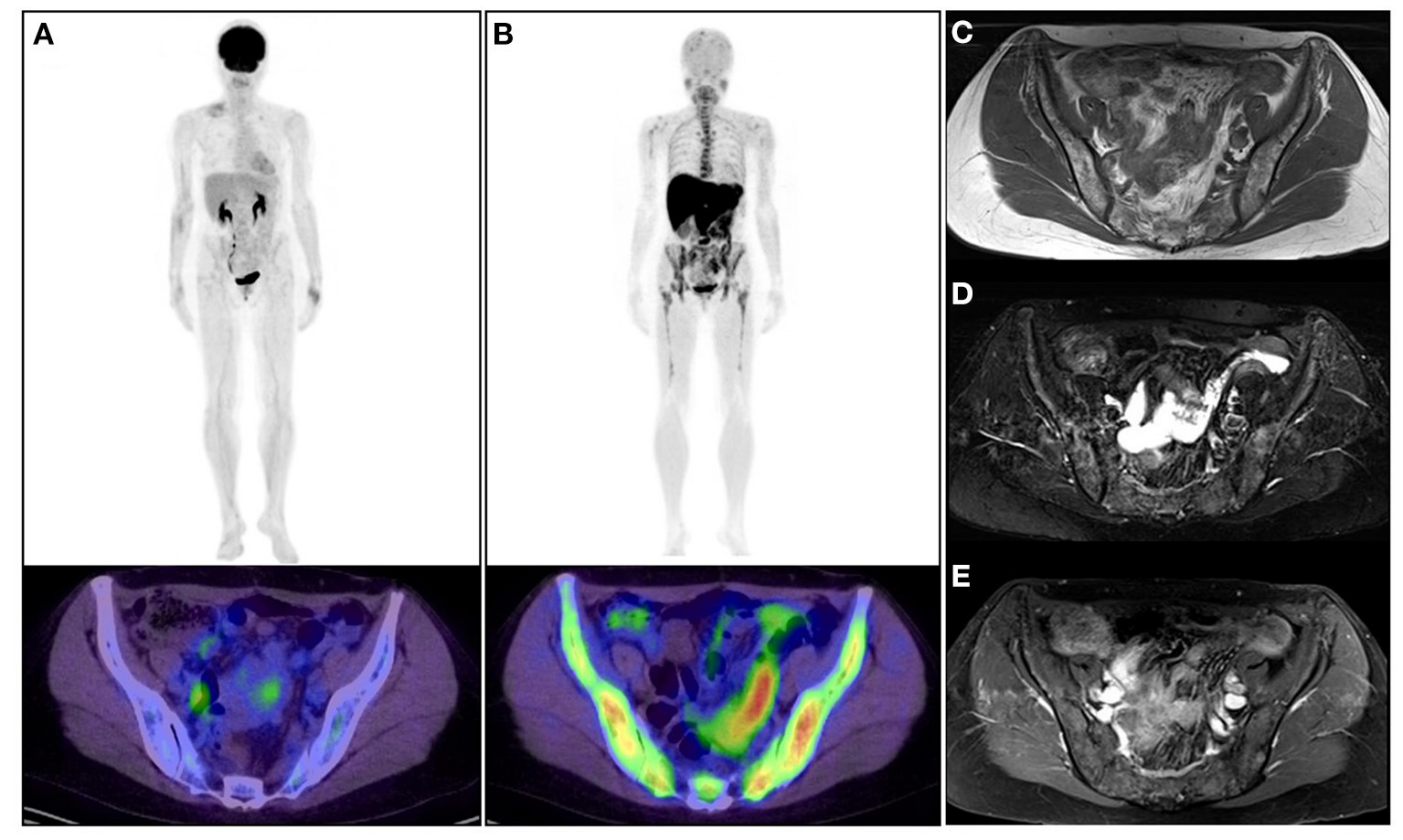
A 53-year-old woman diagnosed with asymptomatic multiple myeloma. **(A)** *Upper*: whole-body 2-[^18^F]-fluoro-2-deoxy-glucose positron emission tomography (^18^F-FDG PET); *lower*: ^18^F-FDG PET/CT image of the pelvis. **(B)** *Upper*: whole-body l-[*methyl*-^11^C]methionine (^11^C-MET) PET; *lower*: ^11^C-MET PET/CT image of the pelvis. **(C)** MRI (TI-weighted image). **(D)** MRI (T2-fat-suppressed image). **(E)** Gadolinium-enhanced image. The patient was observed without any therapeutic intervention. ^18^F-FDG PET/CT and ^11^C-MET PET/CT were performed to confirm the current status of the disease. ^11^*C*-MET PET/CT images represented active diffuse MM lesions in the skeleton (upper and lower extremity, rib, vertebra, and pelvis) much more clearly than ^1^^8^F-FDG PET/CT and MRI. Physiological uptake of ^1^^1^*C*-MET was confirmed in the salivary grand, liver, pancreas, and kidney.

The incorporation of amino acids into newly synthesized immunoglobulins was first reported in 1978 (25). The mechanism of ^11^C-MET uptake in myeloma lesions is the incorporation of methionine into abnormal immunoglobulin production in lesions. The optimal timing for scanning of ^1^^1^*C*-MET PET/CT was 20 min after the administration of ^1^^1^*C*-MET PET/CT. Dankerl et al. (26) reported their initial experience of the visualization of active MM with ^11^C-MET, which was in contrast to its low uptake in the BM. ^11^C-MET could detect both intra- and extramedullary lesions, as we experienced (Figure 1). Nakamoto et al. (27) reported the higher sensitivity of ^11^C-MET PET than ^18^F-FDG (89 vs. 78%) in 20 patients (15 with MM and 5 with plasmacytoma) (27). Lapa et al. (28) presented the significantly higher sensitivity of ^11^C-MET PET than ^18^F-FDG (76 vs. 60%) in the identification of medullary and extramedullary lesions in 78 patients (4 solitary plasmacytomas, 5 SMMs, and 69 symptomatic MMs). In contrast to laboratory data, ^11^C-MET positivity was correlated with the concentrations of β2 microglobulin and free light chains (FLCs), which are key prognostic factors in MM. These findings indicate that ^11^C-MET PET is a promising PET tracer that can indicate disease activity and the degree of BM involvement in MM (28, 29). The representative images of ^11^C-MET PET, which could identify active MM lesions earlier than ^18^F-FDG, are shown in Figure 2.

In the assessment of MM lesions, positive lesions were detected by visual interpretation as areas of locally increased tracer uptake compared with the surrounding normal tissue or contralateral structures (28). Quantitative analysis has been performed using the maximum standardized uptake (SUV_max_) and mean standardized uptake (SUV_mean_) values of the identified MM lesions, in which the SUV_max_ of ^11^C-MET tended to be higher than that of ^18^F-FDG. ^11^C-MET was found to detect more focal MM lesions than ^18^F-FDG (8, 27, 30), and a higher ^11^C-MET uptake was related to poor prognosis (31).

In contrast to the results of visual interpretation of ^11^C-MET-PET in MM, no correlation was found for the semiquantitative parameters (SUV_mean_ and SUV_max_) of ^11^C-MET with cytogenetics or pathological changes in laboratory values, including albumin (<3.5 g/dl), creatinine (>1.2 mg/dl), β2 macroglobulin (>3.5 mg/l), or the levels of free light chains (>100) (29).

A significant correlation was confirmed between BM plasma cell (BMPC) infiltration and the mean target-to-background ratio (TBR_mean_) on ^11^C-MET in the lumbar vertebrae of SMM patients (*r* = 0.789), which is close to that for ^68^Ga-Pentixafor (*r* = 0.724), but higher than that for ^18^F-FDG PET/CT (*r* = 0.355) (31).

In the volume-based analysis, the median total metabolic tumor volume (TMTV) was significantly higher for ^11^C-MET PET/CT than for ^18^F-FDG, with a median difference of 141.2%. In addition, the total lesion ^11^C-MET uptake (TLMU) was higher than the total lesion glycolysis (TLG) of ^18^F-FDG, with a median difference of 216.7% (8). Positive correlations were determined in ^11^C-MET PET/CT for TLMU and the β2 macroglobulin levels, TLMU and the M component, and TLMU and BM infiltration (*r* = 0.450). However, no definite cutoff value has been specified for any quantitative ^11^C-MET uptake value for the assessment of MM; thus, further study is required to estimate a reliable cutoff value.

In approximately 40% of patients, ^11^C-MET PET was superior to ^11^C-choline PET in terms of the number of intramedullary lesions detected (32).

Chemokine receptor-4 (CXC*R4*) plays a crucial role in tumor growth, progression, invasiveness, and metastasis. Overexpression of CXC*R4* was observed in more than 30 different tumors, including MM (33). ^68^*G*a-Pentixafor is a novel PET tracer with high affinity for CXC*R4*. Several studies have demonstrated that ^6^^8^*G*a-Pentixafor was superior or equal to ^1^^8^*F*-FDG PET/CT in the diagnosis of MM (34, 35). By comparing ^6^^8^*G*a-Pentixafor and 11C-MET PET with 18F-FDG PET/CT, the greater sensitivity of ^6^^8^*G*a-Pentixafor and 11C-MET PET/CT in the evaluation of BM involvement in patients with SMM was demonstrated (31).

Uptake of the amino acid tracer fluoro-ethyl-tyrosine (^18^F-FET) in the tumor is mediated by LAT1 and sodium-dependent transport *via* a system similar to system B0 (36–38). Unlike ^11^C-MET, ^18^F-FET is not incorporated into proteins (36, 37).

A basic study showed that the intracellular uptake of ^18^F-FET in a myeloma cell line was lower than that of ^18^F-FDG and ^11^C-MET and that the amount of ^18^F-FET retained by CD138^+^ plasma cells tended to be less than that of ^18^F-FDG and ^11^C-MET (23). A clinical study found sensitivity and specificity values of 55.58 and 9.09%, respectively, for ^18^F-FET PET/CT assessment. Thus, ^18^F-FET does not seem to be a promising biomarker in myeloma imaging (39).

## Proliferation PET for MM

MM is a clonal plasma cell proliferative disorder primary in the BM. The proliferation status of myeloma cells has been assessed using the plasma cell labeling index, nuclear proliferation antigen Ki-67 index, and metaphase cytogenetics (18, 40–43).

[^18^F]Fluorothymidine (^18^F-FLT) is a radiolabeled thymidine analog that is trapped by cells in the S-phase of the cellular cycle (44). It is not incorporated into DNA, thus imaging as a surrogate of Ki-67. ^18^F-FLT PET/CT can indicate proliferation noninvasively in patients with neoplasms. Retention of the tracer within cells partially reflects thymidine kinase activity and is often positively correlated with cellular proliferation (45).

In the first report evaluating ^18^F-FLT PET/CT in MM, the affected osteolytic areas in two MM patients showed low ^18^F-FLT uptake (46). Sachpekidis et al. (47) reported that ^18^F-FLT PET/CT failed to identify myeloma-associated skeletal disease in 60% (3/5) of patients with bone lesions, in contrast to the positive uptake with ^18^F-FDG PET/CT. In addition, the number of identified myeloma lesions was significantly lower with ^18^F-FLT PET/CT than with ^18^F-FDG PET/CT. The SUV_mean_ and SUV_max_ values of MM lesions were significantly higher for ^18^F-FLT than for ^18^F-FDG, but those of normal bone regions were also higher for ^18^F-FLT (47). ^18^F-FLT uptake in the BM of healthy control subjects was increased in the spine (SUV_mean_ = 4.56 ± 1.36) (48), which appears to be a limitation of ^18^F-FLT-PET/CT in differentiating active MM from normal bone. Gallicchio et al. (49) evaluated the prognostic value of the combined use of ^18^F-FDG and ^18^F-FLT PET/CT in patients with MM who had suspected relapse after first-line therapy. The ^18^F-FDG and/or ^18^F-FLT positive group showed worsened event-free survival (EFS) than both negative groups. Therefore, the combined use of ^18^F-FDG and ^18^F-FLT PET/CT in MM can be a prognostic indicator of patients with a higher risk of recurrence.

MM is generally characterized by a low proliferation rate with a very small fraction of proliferating cells (50). The study of Sachpekidis found an increased ^18^F-FLT PET/CT tracer accumulation in a patient with lesions that showed extramedullary expansion. Extramedullary expansion of MM is associated with increased proliferation (51, 52). Thus, ^18^F-FLT appears to be a potential tool for identifying MM patients with a hyperproliferative tumor leading to poor prognosis, but does not seem suitable as a tracer in the diagnosis of MM.

Carbon-11-labeled 4′-thiothymidine (^11^C-4DST) is a thymidine analog and is a cell proliferation imaging agent based on the mechanism of its incorporation into DNA (53–55). ^11^*C*-*4D*ST is synthesized with up to 70% decay-corrected yields (56). Based on clinical investigation, the optimal timing for scanning of ^1^^1^*C*-*4D*ST PET/CT was 20–40 min after its administration (56). All the ^1^^1^*C*-*4D*ST PET/CT studies for the assessment of malignancy in our facility were made consistent to start the PET/CT scan *from 4*0 min after the administration of ^1^^1^*C*-*4D*ST.

^11^C-4DST PET/CT demonstrated positive potential for proliferation imaging in lung cancer and renal cell cancer that correlated highly with Ki-67 (57, 58). The proliferation status estimated from the expression of Ki-67 was associated with advanced stages and prognosis of MM (42), which is why the assessment of MM has been challenged using ^11^C-4DST rather than ^11^C-MET and ^18^F-FDG PET/CT.

**Figure 3:**
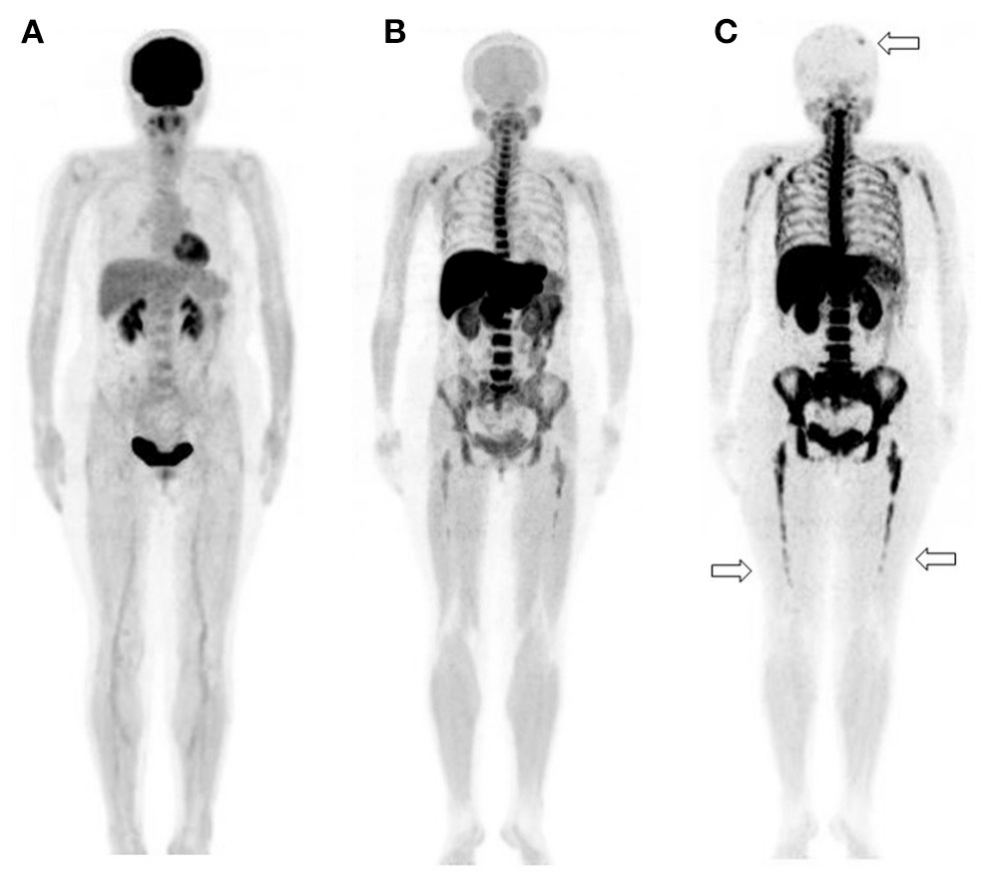
A 65-year-old man diagnosed with IgG-type multiple myeloma was suspected of disease recurrence because of an increased level of myeloma (M) protein. **(A)** 2-[^18^F]-fluoro-2-deoxy-glucose positron emission tomography/computed tomography (^18^F-FDG PET/CT). **(B)** l-[*Methyl*-^11^C]methionine (^11^C-MET) PET/CT. **(C)** Carbon-11-labeled 4′-thiothymidine (^11^C-4DST) PET/CT. ^11^C-MET PET/CT and ^11^C-4DST PET/CT could identify the diffuse active lesions in the bone marrow. Moreover, ^11^C-4DST PET/CT could better detect further disease invasion (*arrow*) than ^11^C-MET PET/CT.

^11^C-4DST PET/CT has higher sensitivity and accuracy than ^18^F-FDG for MM, but similar specificity. There was no statistically significant difference in diagnostic ability between ^11^C-4DST and ^11^C-MET. ^11^C-4DST uptake (15.8 ± 9.79) in untreated MM lesions was higher than the uptake of ^18^F-FDG (4.00 ± 1.63) and ^11^C-MET (5.74 ± 2.15); however, there was little difference in the uptake between ^11^C-MET and ^18^F-FDG for these lesions (30). Compared to the plasma cell infiltration obtained by BM aspiration, ^11^C-4DST has potential for identifying MM patients who have ≥10% plasma cells in the cytology specimen. In the case of 10–20% plasma cells in the cytology specimen, equivocal ^11^C-MET uptake was still confirmed in the MM lesion. All cases of ≥30% plasma cells in the cytology specimen showed positive findings in ^11^C-4DST and ^11^C-MET. In contrast, ^18^F-FDG was positive in the case of ≥59% of plasma cells in the cytology specimen. These results indicate that ^11^C-4DST and ^11^C-MET may be able to detect active MM earlier than is possible with ^18^F-FDG (30). The representative images are shown in Figure 3.

**Figure 4:**
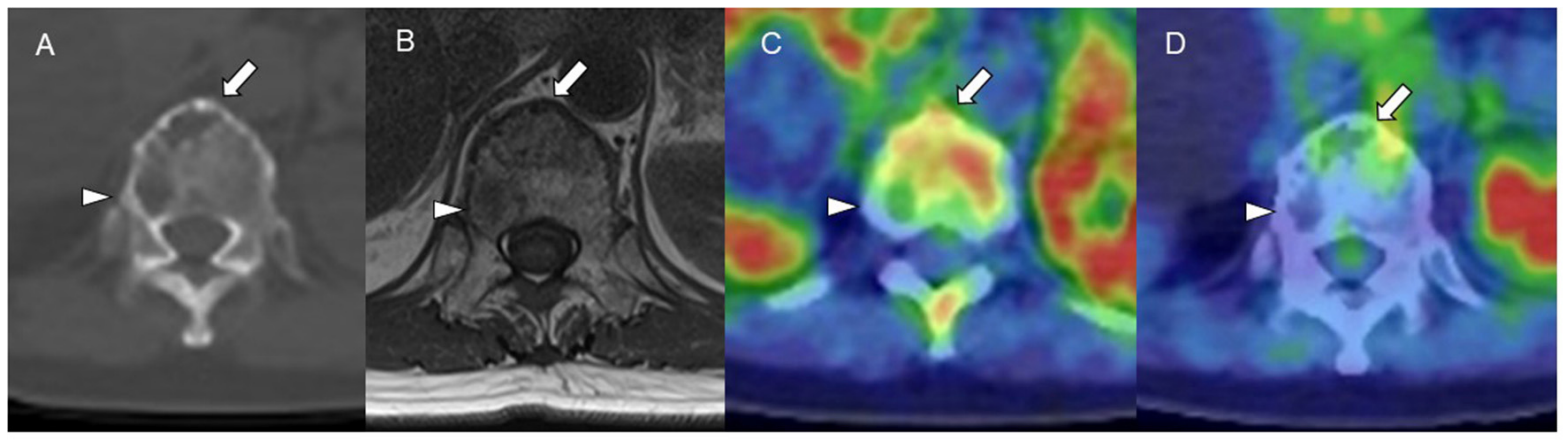
**(A)** CT (bone window). **(B)** MRI (TI-weighted image). **(C)** Carbon-11-labeled 4′-thiothymidine positron emission tomography/computed tomography (^11^C-4DST PET/CT). **(D)** 2-[^18^F]-fluoro-2-deoxy-glucose (^18^F-FDG) PET/CT. ^11^C-4DST could distinguish active (*arrow*) and inactive (*arrowhead*) myeloma lesions in a vertebral body, those that were regarded as all positive on MRI and CT and all negative on ^18^F-FDG.

^11^C-4DST PET/CT may be superior to ^18^F-FDG PET/CT and whole-body magnetic resonance imaging (WBMRI) for the evaluation of MM. Moreover, combined ^11^C-4DST PET and WBMRI showed strong potential for changing the management of patients with MM (59) (Figure 4).

**Figure 5:**
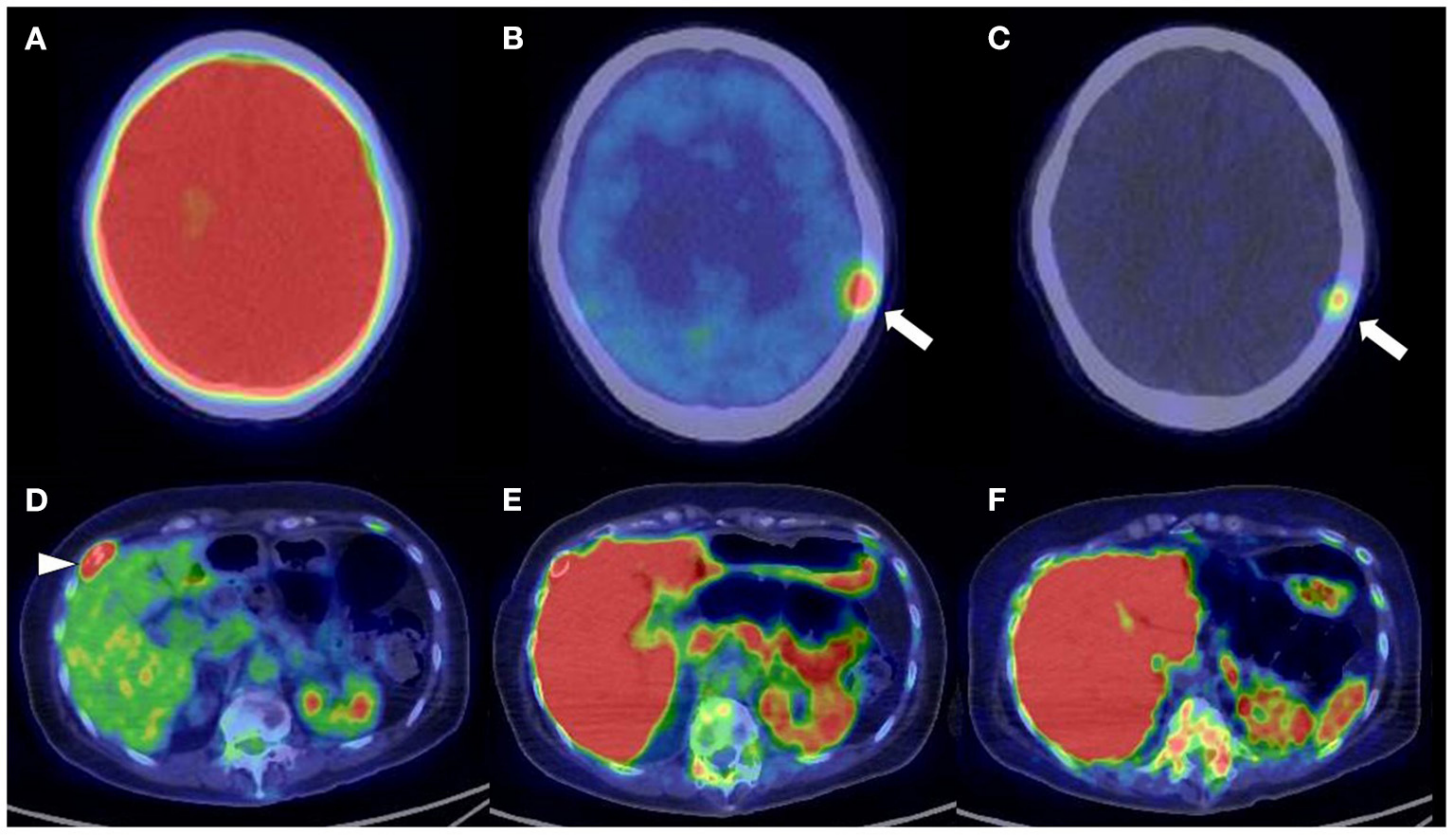
Upper low (cranial bone lesion, *arrow*): **(A)** 2-[^18^F]-fluoro-2-deoxy-glucose positron emission tomography/computed tomography (^18^F-FDG PET/CT). **(B)** l-[*Methyl*-^11^C]methionine (^11^C-MET) PET/CT. **(C)** Carbon-11-labeled 4′-thiothymidine (^11^C-4DST) PET/CT. Lower low (right rib lesion, *arrowhead*): **(D)** ^18^F-FDG PET/CT. **(E)** ^11^C-MET PET/CT. **(F)** ^11^C-4DST PET/CT. Identification of positive lesions close to a high physiological PET uptake can be a limitation of PET scan. In contrast to ^11^C-4DST and ^11^C-MET, which can clearly identify active multiple myeloma (MM) lesions because of their much lower physiological uptake in the brain, ^18^F-FDG PET can miss cranial bone lesions due its high physiological ^18^F-FDG uptake in the brain. ^11^C-4DST and ^11^C-MET are limited for the assessment of lesions close to the liver, which shows high physiological uptake of ^11^C-4DST and ^11^C-MET.

The identification of positive lesions close to a high physiological PET uptake can be a limitation of PET scan. In our experience, ^18^F-FDG PET/CT can miss cranial bone lesions due to the high physiological ^18^F-FDG uptake in the brain; in contrast, ^11^C-4DST and ^11^C-MET can clearly identify active MM lesions because they have much lower physiological uptake in the brain (Figure 4). ^11^C-4DST and ^11^C-MET are limited for the assessment of lesions close to the liver, spleen, pancreas, and kidney, which tend to show high physiological uptake of ^11^C-4DST and ^11^C-MET (Figure 5).

The physiological uptake of ^11^C-4DST in the BM has been estimated as approximately SUV_max_ = 3 (53). ^11^C-4DST uptake in the BM might reflect the underlying activity of MM, estimated by several biomarkers related to its progression (60).

## Challenges in the Diagnosis of Diffuse-Type MM

The skeletal uptake patterns of ^18^F-FDG PET for MM are classified as diffuse BM uptake, focal uptake, mixed (combined diffuse and focal), and normal (61). The Durie–Salmon PLUS staging system applies ^18^F-FDG PET/CT and MRI findings for the staging of MM, with criteria including the number of focal lesions and/or the severity (mild, moderate, and severe) of diffuse disease (62).

A limitation of ^18^F-FDG PET is its unreliability in discriminating diffuse BM involvement from reactive change, with MRI being the modality of choice for imaging diffuse BM involvement (3). Paschali et al. (63) reported that the pelvis/liver SUV_max_ ratio using a cutoff value of 1.1 showed a positive correlation with BM infiltration rate, with specificity of 99% and sensitivity of 76%, indicating its promise as a marker in the assessment of diffuse BM involvement in MM. In our experience, ^11^C-4DST and ^11^C-MET have potential for detecting diffuse BM involvement in MM, possibly at an earlier stage than is possible with ^18^F-FDG. It is necessary to confirm its suitability in a further study.

These promising PET tracers provide a deeper understanding of the molecular mechanism of MM and the status of each patient with MM. Newer PET tracers have the potential of replacing the role of ^18^F-FDG PET/CT in MM due to their better detection of MM lesions; however, they have limitations regarding accessibility and require more solid evidence in further prospective clinical trials.

More sensitive PET tracers for the detection of MM may contribute to assessing the more recent status of patients with MM. However, further studies are required to evaluate the impact of newer PET tracers for the Durie–Salmon PLUS staging system.

## Conclusion

^18^F-FDG PET is established as a standard imaging modality for the assessment of patients with MM. The recently developed PET tracers outperform ^18^F-FDG PET in terms of diagnostic ability and show potential to assist in the selection of the most suitable treatment. However, it is necessary to obtain conclusive evidence in future prospective clinical studies before these findings can be confirmed.

## Author Contributions

RM contributed to the conception or design of the work, reading the existing literature, drafting and checking the paper, and approved the submitted version.

## Conflict of Interest

The author declares that the research was conducted in the absence of any commercial or financial relationships that could be construed as a potential conflict of interest.

## Publisher's Note

All claims expressed in this article are solely those of the authors and do not necessarily represent those of their affiliated organizations, or those of the publisher, the editors and the reviewers. Any product that may be evaluated in this article, or claim that may be made by its manufacturer, is not guaranteed or endorsed by the publisher.
